# Comparison of two chromogenic media: Brilliance Group B and ChromID STRB for the screening of Group B streptococci from an antenatal clinic in Sydney, Australia

**DOI:** 10.1099/acmi.0.001106.v4

**Published:** 2026-07-30

**Authors:** John Merlino, Sophia Rizzo, Daniel Seed, Kimberly Chuen, Olivia Aitkens, Haejin Park, Avani Singh, Steven Siarakas

**Affiliations:** 1 Department of Microbiology and Infectious Diseases, NSW Health Pathology, Concord Hospital, Sydney, Australia; 2 Department of Infectious Disease and Immunology, School of Medical Sciences, Faculty of Medicine and Health, University of Sydney, Sydney, Australia

**Keywords:** Brilliance Group B, Bruker, ChromID STRB, chromogenic media, Group B streptococci, limit of detection, sensitivity, specificity, *Streptococcus agalactiae*

## Abstract

In this study, we compared and assessed the agreement of two commercial chromogenic agars, Brilliance Group B (BGB; Thermo Fisher, Australia) and ChromID STRB (bioMérieux, Australia), in the detection and presumptive identification of Group B streptococci (GBS; *Streptococcus agalactiae*) by colour and later confirmed by MALDI-TOF MS (Bruker Australia). The sensitivity and specificity based on colour were 100 and 91% for BGB and 97 and 100% for ChromID STRB. The NPV (negative predictive value) for GBS on BGB was 100%, and the PPV (positive predictive value) was 73%. ChromID STRB had an NPV of 99% and a PPV of 100%. While both plates compared well in accuracy in the presumptive identification of GBS by colour, both plate media required the integration of MALDI-TOF MS for confirmatory identification. MALDI-TOF MS allowed accurate detection of GBS from both chromogenic plates with a score of >=2.0 after prior treatment with formic acid.

## Data Summary

All data used in this study are available within the manuscript and the attached Excel spreadsheet, Appendix 1 (available with the online version of this article).

## Introduction

Group B streptococci (GBS) identified as *Streptococcus agalactiae* are Gram-positive bacteria found commensally in the human gastrointestinal tract and as part of the vaginal flora of around 15–30% of asymptomatic women [1]. It is notoriously known as a leading cause of sepsis in neonates [2], with early-onset GBS (EOGBS) disease resulting from the vertical transmission of GBS from a colonized mother during labour and delivery [3]. Additionally, EOGBS disease can manifest in neonates as pneumonia and meningitis and overall is associated with high mortality rates of 5–20% and high morbidity rates with 30% of neonates suffering long-term health effects following GBS meningitis [3]. Due to these high rates, it is imperative to identify vaginal carriers of the bacteria. This relies on GBS screening from lower vaginal swabs and should occur at 35–37 weeks' gestation, as vaginal GBS recolonization can occur intermittently during pregnancy [2, 3]. The screening and identification of GBS allow earlier identification and appropriate antibiotic treatment and can be provided to GBS-positive women about to give birth to minimize the risks of EOGBS disease in neonates [4]. Within the literature, the performance of microbiological methods for GBS screening has been greatly improved using selective chromogenic substrates in chromogenic culture media [5]. Studies have shown that chromogenic agar media tested produced comparable results to the pre-enrichment CDC method requiring extended incubation in the isolation, detection and identification of GBS [6]. The aim of this study was to compare and assess the performance of two commercial chromogenic agars, Brilliance Group B and ChromID STRB, in the accuracy of detection and presumptive identification of Group B streptococci by colour from low vaginal swabs received in our laboratory when compared to our MALDI-TOF MS.

## Methods

### Specimens/isolates

A total of 163 antenatal low vaginal swabs were examined for the presence of Group B streptococci on two commercial selective chromogenic media, Brilliance Group B (Thermo Fisher, Australia) and ChromID STRB (bioMérieux, Australia).

### Culture and incubation

The swabs were cultured by direct plating onto Brilliance Group B and ChromID STRB plates in parallel following manufacturer instructions. Chromogenic plates were incubated aerobically at 35 °C in a light-proof box for 24 h and later extended for 48 h to confirm final recovery. Given the sensitivity of previous studies in using these chromogenic selective media, no selective broth culture medium was used in the recovery of GBS [6]. Growth on the chromogenic plates was classified as light pink (24 h) or purple (48 h) in colour on Brilliance Group B plates and pink-red colonies on ChromID STRB plates. No growth on each agar was a negative result. Growth of any colony colour was further investigated, and organisms were identified by MALDI-TOF MS. Identification other than GBS was considered a negative result (Appendix 1). Growth of any other species was considered to be a negative result. Results of each plate read were determined by two separate investigators blindly, and the data were later collated by a third investigator. Control strain *S. agalactiae* ATCC 12236 was used as a positive control, and *Enterococcus faecalis* ATCC 29212 was used as a negative control to assess chromogenic colour and batch quality control.

The limit of detection of Brilliance Group B plates and ChromID STRB plates was determined by performing several 10-fold dilutions from 1.5×10^8^ c.f.u. l^−1^ (0.5 McFarland) suspension using ATCC 12236 *S. agalactiae* as the control organism. Each dilution was plated, and growth was recorded.

### Identification

Any presumptive positive colonies at 24 h or 48 h were confirmed using MALDI-TOF MS (MALDI Biotyper Sirius One MBT IVD Library Revision J 2022, Bruker, Australia) following manufacturers' recommendations with prior treatment of isolates with formic acid. This was used as the final gold standard for identification for all presumptive isolated colonies.

### Statistical analysis

Analysis of the results by colour included determining the positive and negative predictive value for each chromogenic plate media, as well as determining their sensitivity and specificity in accuracy in identifying and detecting GBS by colour compared to the MALDI-TOF MS as the gold standard with a score >2.0. MedCalc easy-to-use statistical software was used to calculate comparable differences and 95% confidence limits (https://www.medcalc.org/en/calc/diagnostic_test.php).

## Results

Results are shown in Table 1. Results were analysed by the online MedCalc easy-to-use statistical software. Chromogenic findings were analysed and compared to the results of MALDI-TOF MS identification as the gold standard. Both chromogenic media gave a positivity rate of 20.25% when compared to MALDI-TOF MS identification. Brilliance Group B chromogenic media gave a sensitivity of 100% with a 95% CI of 89.42–100% when compared to ChromID STRB with a sensitivity of 97% with a 95% CI of 84.24–99.92%. The specificity was better with ChromID STRB with a value of 100% with a 95% CI of 97.20–100% when compared to Brilliance Group B with a specificity of only 91% with a 95% CI of 84.43–95.14%, mainly due to an increased number of false-positive bacterial strains confirmed with a MALDI-TOF MS identification score of >=2.0. The PPV was much better for ChromID STRB with a value of 100% with a 95% CI of 89.11–100% and an NPV of 99.24% with a 95% CI of 94.97–99.89% when compared to Brilliance Group B with a PPV of only 73% with a 95% CI of 61.60–82.50% and an NPV of 100% with a 95% CI of 94.97–99.89%. Due to fewer false positives, ChromID STRB had a higher accuracy of 99.36% with a 95% CI of 96.63–100% when compared to Brilliance Group B of 92.64% with a 95% CI of 87.49–96.14%.

**Table 1.** The agreement of the two chromogenic agars to detect and identify GBS by colour confirmed by MALDI-TOF MS

Media	Sensitivity %	Specificity %	PPV %	NPV %	95% CI %	Accuracy %	95% CI %
**Brilliance GBS**	100	91	73	100	61.60 to 82.50	92.64	87.49 to 96.14
**ChromID STRB**	97	100	100	99.24	94.97 to 99.89	99.36	96.63 to 99.98

95% CI (‘exact’ Clopper–Pearson CI ratios).

GBS, Group B streptococci.; NVP, negative predictive value; PPV, positive predicted value.

Both Brilliance GBS plates and ChromID STRB had a limit of detection of 1.5×10^2^ c.f.u. l^−1^ of organism using *S. agalactiae* ATCC 12236 as a control organism (Table 2).

**Table 2.** Brilliance GBS and ChromID STRB chromogenic plates: limit of detection with *S. agalactiae* ATCC 12236

					c.f.u. l^−1^			
**Dilution factor**	0.5 MCF	01 : 10	1 : 10^−2^	1 : 10^−3^	1 : 10^−4^	1 : 10^−5^	1 : 10^−6^	1 : 10^−7^
	1.5×10^8^	1.5×10^7^	1.5×10^6^	1.5×10^5^	1.5×10^4^	1.5×10^3^	1.5×10^2^	1.5×10
**Brilliance GBS**	Growth	Growth	Growth	Growth	Growth	Growth	No growth	No growth
**ChromID STRB**	Growth	Growth	Growth	Growth	Growth	Growth	No growth	No growth

## Discussion

In comparison, both Brilliance GBS plates and ChromID STRB plates had comparable sensitivity and specificity in accurately presumptively identifying GBS. Previous comparable studies have yielded mixed sensitivity results, with other studies [7, 8] also having found the plates to be 100%, while Thermo Fisher Scientific’s own research found the Brilliance GBS plate to have a sensitivity rate of 94.4% [7]. In this study, Brilliance Group B had a false positivity rate of 9%, while ChromID STRB had a false negativity rate of 3%. False-positive bacterial species on Brilliance Group B included *Streptococcus gordonii*, *Streptococcus oralis*, *Lactobacillus salivarius*, *Streptococcus vestibularis*, *Corynebacterium simulans* and *Streptococcus anginosus*. This made Brilliance Group B more labour-intensive since it required the identification of these species. Inhibition of normal flora is important when single colonies are required for MALDI-TOF MS identification or when other work-up is required, including purity for antibiotic susceptibility testing directly from isolated colonies at 24 or 48 h incubation.

In summary, these two selective chromogenic plates offer the laboratory a streamlined screening workflow from swab to result in detecting Group B streptococci by colour. Both plates were found to be comparable and suitable for the detection of Group B streptococci from antenatal swabs by colour with high accuracy. However, both chromogenic agars required the integration of MALDI-TOF MS for final confirmatory identification of presumptive positive colonies prior to organism reporting.

## Supplementary material

10.1099/acmi.0.001106.v4 Supplementary Material 1.

## Abbreviations

ATCC: American Type Culture Collection
BGB: Brilliance Group B
EOGBS: early-onset GBS
GBS: Group B streptococci
IVD: in vitro diagnostics
MBT: MALDI Biotyper
NPV: negative predictive value
PPV: positive predicted value
PPV: positive predicted value

## References

[1] Tazi A, Disson O, Bellais S, Bouaboud A, Dmytruk N (2010). The surface protein HvgA mediates group B *Streptococcus* hypervirulence and meningeal tropism in neonates. J Exp Med.

[2] Larsen JW, Sever JL (2008). Group B *Streptococcus* and pregnancy: a review. Am J Obstet Gynecol.

[3] Mahieu L, Langhendries J-P, Cossey V, De Praeter C, Lepage P (2014). Management of the neonate at risk for early-onset Group B streptococcal disease (GBS EOD): new paediatric guidelines in Belgium. Acta Clin Belg.

[4] Verani JR, McGee L, Schrag SJ (2010). Division of bacterial diseases, national center for immunization and respiratory diseases, centers for disease control and prevention (CDC). Prevention of perinatal group B streptococcal disease: revised guidelines from CDC, 2010. MMWR Recomm Rep.

[5] Morita T, Feng D, Kamio Y, Kanno I, Somaya T (2014). Evaluation of chromID strepto B as a screening media for *Streptococcus agalactiae*. BMC Infect Dis.

[6] Salem N, Anderson JJ (2015). Evaluation of four chromogenic media for the isolation of group B *Streptococcus* from vaginal specimens in pregnant women. Pathology.

[7] Verhoeven PO, Noyel P, Bonneau J, Carricajo A, Fonsale N (2014). Evaluation of the new brilliance GBS chromogenic medium for screening of *Streptococcus agalactiae* vaginal colonization in pregnant women. *J Clin Microbiol*.

[8] Clarke D, Scopes E (2013). Thermo scientific brilliance GBS agar for group B Streptococcus screening. https://assets.thermofisher.com/TFS-Assets/MBD/posters/Thermo-Scientific-Brilliance-GBS-Agar-For-Group-B-Streptococci-Screening-GLOBAL-LT2079-EN-low-res.pdf.

